# 69 Beyond motivation: Essential factors for implementing lifestyle interventions in community care to enhance physical activity among community dwelling older adults

**DOI:** 10.1093/eurpub/ckae114.008

**Published:** 2024-09-26

**Authors:** Patricia Van Der Laag, Berber Dorhout, Cindy Veenhof, Di-Janne Barten, Lisette Schoonhoven

**Affiliations:** Julius Center for Health Sciences and Primary Care, Nursing Science, University Medical Center Utrecht, Utrecht, The Netherlands; Research Group Innovation of Human Movement Care, Research Centre for Healthy and Sustainable Living, Utrecht University of Applied Sciences, Utrecht; Research Group Innovation of Human Movement Care, Research Centre for Healthy and Sustainable Living, Utrecht University of Applied Sciences, Utrecht; Department of Rehabilitation, Physical Therapy Science & Sports, University Medical Center Utrecht, Utrecht University, Utrecht; Center for Physical Therapy Research and Innovation in Primary Care, Julius Health Care Centers, Utrecht; Research Group Innovation of Human Movement Care, Research Centre for Healthy and Sustainable Living, Utrecht University of Applied Sciences, Utrecht; Department of Rehabilitation, Physical Therapy Science & Sports, University Medical Center Utrecht, Utrecht University, Utrecht; Julius Center for Health Sciences and Primary Care, Nursing Science, University Medical Center Utrecht, Utrecht, The Netherlands; Faculty of Health Sciences, University of Southampton, Southampton

## Abstract

**Purpose:**

ProMuscle is a lifestyle intervention that combines protein-enriched nutrition and resistance-exercise training. It is effective in improving physical functioning in community-dwelling older adults and contributes to the maintainance of independency. Unfortunately, ProMuscle’s potential is impeded by insufficient attention to implementation. The PromUscle iMPlementation study (PUMP-fit) proposes to provide knowledge and guidance to healthcare professionals and policymakers for successful implementation of ProMuscle in multiple community-care settings.

**Project description:**

The PUMP-fit study focuses on implementing ProMuscle in multiple community-care settings in the Netherlands. We employed a longitudinal mixed-methods design. Initially, a scoping review identified barriers and facilitators for implementation. Subsequently, in co-design with stakeholders like physiotherapists, dieticians, implementation experts, and older adults feasible, practical and theory-based implementation strategies (targeting knowledge, collaboration, patient needs and costs) were developed and processed into an online implementation toolbox. Recently, a hybrid type III stepped-wedge cluster trial was conducted to evaluate the implementation toolbox’ effectiveness. In addition to longitudinal insights into implementation outcomes like the adoption and fidelity of ProMuscle, patient outcomes were collected to evaluate ProMuscle’s effect on physical functioning. With the results of the PUMP-fit study we will inform policy makers and healthcare professionals how to support implementation of lifestyle interventions in community-settings.

**Conclusions:**

ProMuscle is an effective intervention to improve community-dwelling older adults’ physical functioning. The developed implementation toolbox is a feasible way to support healthcare professionals during implementation. However, implementation is not self-evident. As confirmed in this study, multiple factors such as, insufficient time, money, organizational facilitation, and high intervention costs, negatively influences the implementation and lead to low recruitment rates. These implementation challenges cannot always be eliminated by an individual professional, even though professionals receive individual implementation support. Therefore, support from relevant stakeholders like policy makers is necessary. For example, to raise awereness about the importance of exercise and nutrition among older adults, policymakers, and healthcare professionals. Additionally, it is suggested that a uniform payment system could contribute to the implementation of lifestyle interventions to enhance physical actvity in community-dwelling older adults. The implementation toolbox and policy manual will be freely available and distributed through various communication channels for further upscaling.

